# Clinical relevance of positive intraoperative bacterial culture in tibial plateau leveling osteotomy in dogs: a retrospective study

**DOI:** 10.1186/s12917-024-04007-w

**Published:** 2024-05-04

**Authors:** Natália Korytárová, Sabine Kramer, Oliver Harms, Holger A. Volk

**Affiliations:** Department of Small Animal Medicine and Surgery, University of Veterinary Medicine Hannover, Bünteweg 9, 30559 Hannover, Germany

**Keywords:** Tibial plateau leveling osteotomy, Intraoperative bacterial culture, Surgical site infection

## Abstract

**Background:**

Tibial plateau leveling osteotomy (TPLO) belongs to the most frequently used surgical method for the treatment of cranial cruciate ligament rupture in dogs. Surgical site infection (SSI) is one of the possible postoperative complications. The aim of this study was to evaluate the diagnostic value of intraoperative bacterial culture as a tool for the detection of intraoperative bacterial contamination progressing to infection development in canine TPLO. Electronic patient records from dogs who underwent TPLO between January 2018 to December 2020 were retrospectively reviewed. Intraoperative bacterial culture results, used antimicrobial drugs and presence of SSI were recorded.

**Results:**

Ninety-eight dogs were included in the study. SSI rate was 10.2%. All dogs who developed SSI (*n* = 10) had negative intraoperative bacterial cultures. None of the dogs with positive intraoperative bacterial culture (*n* = 6) developed SSI. The most cultured bacteria causing SSI was *Staphylococcus pseudintermedius* (*n* = 4).

**Conclusions:**

Intraoperative bacterial culture in dogs undergoing TPLO is not suitable as a predictor of surgical site infection.

## Background

Cranial cruciate ligament disease is one of the main reasons for pelvic limb lameness in dogs [1]. Currently, one of the most common methods of surgical treatment of canine cranial cruciate ligament rupture is tibial plateau leveling osteotomy (TPLO) [2–6].

The complication rate of TPLO varies between 10 and 34%, with 2 to 4% requiring surgical revision.[7] Surgical site infection (SSI) rate after TPLO is reported to be 2.9–25.9% [8–29], which is higher compared to other clean orthopaedic surgeries (2.0-6.7%) [28, 30, 31]. A complicating factor in the treatment of surgical site infections after an implant surgery like TPLO is the formation of a biofilm, which makes eradication of the infection problematic [32–34]. In some cases, it is possible to defeat the infection with long-term administration of antimicrobial drugs, but usually surgical removal of the implant is necessary [11, 24, 32, 33, 35]. Therefore treatment associated with SSI after TPLO often requires considerable financial costs [36, 37]. Because of the high morbidity due to further surgery and the additional cost in case of a SSI post-TPLO, strategies are being sought to prevent infection development [27].

Early detection and treatment of bacterial contamination of the surgical site can reduce the incidence of SSI [38]. Intraoperative bacterial cultures have been collected for the identification of bacterial contamination in people and dogs undergoing total hip replacement [38–41]. In one study, isolation of *Staphylococcus aureus* from intraoperative bacterial culture in people undergoing total hip replacement was associated with a 7-fold increased risk of infection [40]. On the contrary, another study in people did not find any association between positive intraoperative bacterial culture and SSI development [39]. In canine patients undergoing total hip replacement, positive intraoperative bacterial culture was found not to be a predictor of SSI [38, 41].

To the authors’ knowledge, no study has been conducted on the usefulness of intraoperative bacterial culture taken during canine TPLO. The aim of the current study was to evaluate the clinical relevance of positive intraoperative bacterial culture in dogs undergoing TPLO. We hypothesized that the development of surgical site infection would not be associated with positive intraoperative bacterial culture.

## Methods

This study was performed at the Department of Small Animal Medicine and Surgery, University of Veterinary Medicine Hannover, Germany. All electronic patient records from dogs undergoing TPLO in a three-year period, from January 2018 to December 2020, were evaluated retrospectively. In these patients, breed, age, gender, weight and administered antimicrobial drugs were documented. Results of bacterial culture swabs taken intraoperatively and the occurrence of surgical site infection were recorded. In cases of SSI, results of bacterial culture taken from the infected surgical site were also recorded.

### Diagnostics and surgery

For a non-invasive evaluation of the intraarticular structures of the stifle joint, magnetic resonance imaging (MRI) was performed. The diagnostic part and the surgery were performed either in two independent anaesthetic sessions or in one appointment according to the owner’s wish. All patients were prepared for surgery following the standard aseptic preparation protocol used at the hospital [42]. In patients with a meniscal lesion, a medial arthrotomy followed by partial meniscectomy was performed. TPLO was then performed as described by Slocum and Slocum [43]. After the surgery, the surgical site was covered by a sterile wound dressing applied in the surgical theatre using sterile technique.

### Bacterial culture sampling

Sampling was performed before wound closure. A swab was taken from the surgical site, from the area around the placed TPLO plate and adjacent soft tissues. All samples were stored cooled in Amies transport medium and sent to the laboratory on the day of sample collection. All samples were examined in an accredited microbiological laboratory, in the Institute of Microbiology, University of Veterinary Medicine Hannover. Samples were processed immediately upon arrival at the laboratory. Swabs were streaked onto Columbia Sheep Blood Agar, Boiled Blood Agar and Schaedler Agar. Then the swabs were immersed in nutrient broth. In all cases, both aerobic and anaerobic culture was performed.

### Postoperative management

All dogs remained hospitalized in the clinic until the next day when the wound dressing was changed. Contact with the surgical site was carried out only with single-use examination gloves. The dogs had to wear an Elizabethan collar to prevent licking of the wound. All dog owners were informed by the time of discharge about postoperative wound management. They also received this information on the discharge documents. After receiving the bacterial culture results, which was usually 7 days after the surgery, the owners were contacted by phone and asked about the general condition of the patient and wound healing. This was recorded and considered in our study. Referring veterinarians were asked to contact the clinic in case of any complications. Control radiographs were performed at our clinic six weeks after surgery. Dogs who developed any complications were seen back earlier. Medical records of the dogs in the study were followed up for one year after the surgery. Dogs with incomplete data were excluded from the study.

### Definition of SSI

The definition of SSI was adapted from the standard criteria developed by the US Centers for Disease Control and Prevention (CDC) [44, 45]. A wound was considered infected when purulent discharge, an abscess, or a fistula and/or one or more of the clinical signs of pain and localized swelling, redness, heat, fever, or deep incision spontaneous dehiscence was identified on clinical examination and/or when an organism was isolated from an aseptically collected sample by culture and/or positive cytology study. SSIs were classified according to superficial, deep, or organ/space infections (Table 1). Cases with positive intraoperative bacterial culture but without any clinical signs of infection, were not considered infected.

**Table 1:** Criteria for defining a surgical site infection [44]

	Superficial incisional SSI	Deep incisional SSI	Organ/space SSI
Timing	Within 30 days of surgery	Within 30 days of surgery or 1 year if implant in place	Within 30 days of surgery or 1 year if implant in place
Location	Only skin or subcutaneous tissues of the incision	Deep soft tissues (i.e. fascial and muscle layers) of the incision	Any area other than the incision which was opened or manipulated during surgery
Clinical aspects (one or more must be present.	– Purulent discharge – Organisms isolated from an aseptically collected sample of fluid or tissue – One or more of pain or tenderness, localized swelling, redness, heat and incision is deliberately opened by surgeon unless culture negative	– Purulent drainage from the deep incision but not organ/space – Deep incision spontaneously dehisces or is deliberately opened when patient has one or more of fever, localized pain or tenderness unless culture negative – Abscess or other evidence of infection on direct exam, during re-operation or by histopathology or radiology	– Purulent drainage from drain that is placed into the organ/space – Organisms isolated from aseptically collected sample from organ/space – Abscess or other evidence of infection on direct exam, during re-operation or by histopathology or radiology – Diagnosis of organ/space SSI by attending clinician

### Data analysis

All data were transferred from the clinic’s electronic practice management software Easyvet (Veterinärmedizinisches Dienstleistungszentrum (VetZ) GmbH, Isernhagen, Germany), where they were originally documented, into a spreadsheet in Excel (version 2021, Microsoft, Redmond, Washington, USA) and then imported in a statistical software for further analysis. All analyses were performed with SPSS Statistics 20 (IBM, Armonk, NY, USA). The very low number of positive bacterial cultures observed restricted statistical test procedures and therefore the data were analysed with descriptive statistics only.

## Results

Ninety-eight dogs met the inclusion criteria. Among the 98 dogs, there were 26.5% (26) spayed females, 24.5% (24) intact females, 24.5% (24) intact males and 24.5% (24) neutered males. The median age in years was 5 (range 1–13). A total of 36 different dog breeds were included. Mixed breed dogs were most common (22), followed by Labrador Retriever (10), Golden Retriever (6), American Staffordshire Terrier (4), Boxer (4), Rottweiler (4) and Siberian Husky (4). The median body weight in kg was 32.7 (range 11.5–63.0).

### Surgical procedure

51% (50/98) of the surgical procedures were performed on the left pelvic limb and 49% (48/98) on the right pelvic limb. In 58.2% (57/98) of cases, an MRI was performed prior to surgery. In 41.8% (41/98) dogs, the diagnostic imaging and surgery were split into two separate anaesthetic sessions. Medial arthrotomy and partial meniscectomy were performed in 38.8% (38/98) of dogs.

### Antimicrobial drug use

Prophylactic perioperative antimicrobial therapy was administered in all 98 dogs. Either cefazolin (22 mg/kg IV) or amoxicillin/clavulanic acid (12,5 mg/kg IV) were administered. Cefazolin was administered in 65.3% (64/98) dogs and 34.7% (34/98) patients received amoxicillin/clavulanic acid. All 98 dogs received amoxicillin/clavulanic acid (12,5 mg/kg PO q 12 h) postoperatively. The median duration of the postoperative antimicrobial therapy was 7 days (range 5–28 days).

### Intraoperative bacterial culture

Intraoperative bacterial culture was collected in all 98 dogs. 93.9% (92/98) of dogs had negative intraoperative bacterial culture. Bacteria were isolated in only six dogs (6.1%). In all cases with positive intraoperative bacterial culture, there was only low bacterial contamination detected. None of these six dogs developed a surgical site infection. Summary of the positive intraoperative bacterial culture results is shown in Table 2.

**Table 2:** Summary of the positive intraoperative bacterial culture results in dogs undergoing TPLO

	Intraoperative bacterial culture	Bacterial count	Postoperative antimicrobial therapy	Susceptibility to amoxicillin/clavulanic acid	SSI
1.	*Micrococcus luteus*	low	amoxicillin/clavulanic acid	sensitive	no
2.	*Pseudomonas spp.*	low	amoxicillin/clavulanic acid	sensitive	no
3.	*Pseudomonas spp.*	low	amoxicillin/clavulanic acid	sensitive	no
4.	*Staphylococcus pseudintermedius*	low	amoxicillin/clavulanic acid	sensitive	no
5.	*Staphylococcus hominis*	low	amoxicillin/clavulanic acid	resistant	no
6.	*Staphylococcus hominis*	low	amoxicillin/clavulanic acid	sensitive	no

### Surgical site infection

Surgical site infection was diagnosed in a 10.2% (10/98) of dogs. All dogs, who developed a SSI, had a negative result in the intraoperative bacterial culture. Seven dogs developed a superficial SSI and three dogs a deep SSI. All patients with superficial SSI were treated only medically. Medical therapy consisted of systemic antimicrobial treatment combined with local antiseptic therapy using wound irrigation solution (Prontovet, B Braun, Melsungen, Germany). Implant removal was performed in all three dogs with deep SSI. Bacterial culture sampling from the infected surgical site was performed in all three dogs with deep SSI and in two dogs with superficial SSI. In the remaining five dogs with superficial SSI, no bacterial culture sampling was performed. The most cultured bacteria causing SSI in our study was *Staphylococcus pseudintermedius* (4), followed by *Staphylococcus aureus* (1) and *Pseudomonas spp*. (1). In one patient a polymicrobial culture was identified. The summary of the cultured bacterial strains in dogs with SSI after TPLO is shown in Table 3.

**Table 3:** Summary of the cultured bacterial isolates in dogs with surgical site infection after TPLO

	Intraoperative bacterial culture	Surgical site infection	Bacterial culture from the infected wound	Bacterial count	Susceptibility to amoxicillin/clavulanic acid
1.	negative	superficial	no bacterial culture performed	-	-
2.	negative	deep	*Staphylococcus pseudintermedius*	moderate	sensitive
3.	negative	superficial	no bacterial culture performed	-	-
4.	negative	superficial	no bacterial culture performed	-	-
5.	negative	deep	*Staphylococcus pseudintermedius*	low	sensitive
6.	negative	superficial	no bacterial culture performed	-	-
7.	negative	superficial	*Staphylococcus aureus*	moderate	sensitive
8.	negative	superficial	*Staphylococcus pseudintermedius*	low	resistant
9.	negative	superficial	no bacterial culture performed	-	-
10.	negative	deep	*Staphylococcus pseudintermedius* *Pseudomonas spp.*	low low	resistant sensitive

## Discussion

In our study, intraoperative bacterial culture was positive in six cases. None of these six dogs developed a SSI. Conversely, some of the dogs with a negative intraoperative bacterial culture later developed SSI. This finding suggests that positive intraoperative bacterial culture in TPLO patients is not an accurate predictor of surgical site infection. Therefore, our initial hypothesis can be accepted. The same finding was documented in both veterinary studies evaluating the clinical relevance of intraoperative bacterial cultures in canine total hip replacement [38, 41]. In people, the combination of a positive opening and a positive closing culture was a significant predictor of subsequent infection [39, 40]. In our study, only one intraoperative bacterial culture was taken at the end of the surgery before wound closure. Further studies are needed to determine whether the collection of two samples, at the beginning and at the end of surgery, with a positive finding can predict the development of SSI in dogs after TPLO.

There are several possible reasons why the contamination of the surgical site did not result in SSI. It is possible that the bacterial contamination was suppressed by the administration of antimicrobial drugs in the postoperative period. The cultured isolates were sensitive to the administered antimicrobial drug, except in one case. Although resistant bacteria were found in one patient, no SSI developed. Therefore, the question whether postoperative antimicrobials played a role in suppressing bacterial contamination or whether patients would have coped with low bacterial contamination even without the use of antimicrobials in the postoperative period remains unclear. Another reason could be low pathogenicity of the cultured bacteria or the presence of a subclinical infection.

Development of a surgical site infection is an inherent risk in orthopaedic surgery [45]. The SSI rate in this study was 10.2%, which is within the range of previously reported results [8–29]. In the current study, SSI developed in 10 cases. All dogs with SSI had a negative intraoperative bacterial culture. It is highly probable that these infections occurred after the surgery. Medical personnel, environment and commensal organisms from the patient’s own microbiome are potential sources for surgical site contamination [46]. Hands of the medical workers represent an important role in the infection development in the early postoperative period [45, 46]. In the study from Anderson et al. (2014) the use of hand hygiene products at veterinary clinics was often lower than recommended and the overall hand hygiene compliance was poor [47]. It is important that hands are washed or disinfected before and after contact with every patient and wound dressings are used after surgery to reduce the exposure to possible exogenous contamination sources [45]. To prevent contamination of the surgical site during hospital stay, a sterile plaster was applied on the wound after surgery and any handling with the wound was performed using single-use examination gloves. The owners of the dogs were instructed how to properly handle the surgical wound and the dogs had to wear Elizabethan collar to prevent wound licking. Nevertheless, based on our results, it seems that surgical site contamination in our patients occurred in the postoperative period. It remains unclear whether the clinic environment became the source of contamination or whether the contamination only occurred in the home environment after discharge from the hospital. Although we did not find any mention in the reviewed medical records of dogs licking their wounds, we cannot rule this out with certainty.

The most frequently cultured organisms causing SSI in this study were bacteria *Staphylococcus pseudintermedius*. In many other recent TPLO studies, *Staphylococcus spp*. predominated as the cause of surgical site infection as well [11, 19, 20, 22–25, 27, 29, 48]. Staphylococci are skin commensals and opportunistic pathogens, which probably explains their frequent occurrence in surgical site infections [11, 46]. Their significant feature is the ability to form resistance to antimicrobial drugs [49]. The most relevant species include coagulase-positive species *Staphylococcus aureus* and *Staphylococcus pseudintermedius *[49]. Other bacterial strains commonly causing SSI in veterinary orthopaedic surgery are *Streptococcus spp*., *Enterococcus spp*. and *Pseudomonas spp. *[10, 11, 20, 24, 27, 35].

Prophylactic perioperative administration of antimicrobial drugs is a well-established tool to prevent surgical site infection [46]. Opinions on the role of postoperative antimicrobial drugs in dogs after TPLO differ [36]. There are several studies supporting the use of postoperative antimicrobial drugs in TPLO patients [12–15, 20, 23, 26, 50]. However, there are also some reports in which postoperative antimicrobial therapy in TPLO patients is not considered beneficial [10, 16, 19, 22, 27]. The first review article on postoperative antimicrobial drug use after TPLO from Budsberg et al. (2021) concluded that there is little evidence to support protective effect of postoperative antimicrobials against the development of surgical site infection in dogs after TPLO. Nevertheless, the answer to this question from a clinical point of view remains unclear due to only a small number of prospective studies and inconsistent treatment protocols in the reviewed studies [36]. All dogs in our study received antimicrobial drugs postoperatively. Most of the cultured organisms were sensitive to the antimicrobials used. Resistant bacteria were isolated only in one dog with intraoperative contamination and in two dogs with SSI. It is possible that if antimicrobials were not administered in the postoperative period, the incidence of surgical site infection would be higher.

The main limitation of this study was its retrospective nature. It is possible that mild infections, particularly more superficial ones, which resolved without any medical intervention, were not identified or reported. Therefore, the number of cases with SSI could have been underestimated. Conducting our study in a prospective fashion with a uniform antibiotic protocol and bacteriological sampling in all cases of SSI would increase the power of our results. Due to the fact that taking bacterial culture swabs is associated with a certain rate of false negative results, taking a tissue sample for culture would be preferable. Another limitation of the study was the low number of positive intraoperative cultures. A higher number of positive findings would enable us to perform statistical analysis which would increase the power of this study.

## Conclusions

Based on our results, intraoperative bacterial culture does not seem to be a suitable method to predict infection development in dogs undergoing TPLO and it is uncertain whether cultured organisms can cause infection at all. We assume that the contamination of the surgical site and subsequent infection occurred in our patients in the postoperative period and therefore adherence to the hygiene principles in the postoperative period remains an important part in the fight against surgical site infection.

## Data Availability

The data that support the findings of this study are available on request from the corresponding author. The data are not publicly available due to privacy or ethical restrictions.

## Abbreviations

TPLO: Tibial plateau leveling osteotomy
SSI: Surgical site infection
MRI: Magnetic resonance imaging
CDC: US Centers for Disease Control and Prevention
